# Artificial Intelligence for Diagnosis, Risk Stratification, and Prognosis of Neuroblastoma - A Systematic Review and Meta-Analysis

**DOI:** 10.1007/s11912-026-01824-0

**Published:** 2026-09-04

**Authors:** Daniel Rodrigo Serbena, Isabela Luiza Fraron Cieslack, Renan Cassiano Ratis, Débora Van Putten Chaves, Sergio Servilha de Oliveira Filho, Henrique Neves, Fernando Sluchensci dos Santos, Daniel Roberto Cassar, Weber Claudio da Silva, Juliana Sartori Bonini

**Affiliations:** 1 Department of Medicine, Universidade Estadual do Centro Oeste, Guarapuava, Paraná Brazil; 2 Department of Pharmacy, Universidade Estadual do Centro Oeste, Guarapuava, Paraná Brazil; 3 Ilum School of Science, Brazilian Center for Research in Energy and Materials (CNPEM), Campinas, Sao Paulo 13083-970 Brazil; 4 Department of Medicine, Universidade Federal do Paraná, Curitiba, Paraná Brazil

**Keywords:** Machine Learning, Risk Stratification, Differential Diagnosis, Prognosis, MYCN, Cancer

## Abstract

**Purpose:**

To synthesizes evidence on artificial intelligence (AI) performance in neuroblastoma (NB) diagnosis, risk stratification, prognosis, and genomic characterization.

**Materials and methods:**

A systematic review and meta-analysis was conducted following PRISMA 2020 guidelines (PROSPERO: CRD42024539475) across five databases. Meta-analyses used random-effects models with logit-transformed Area Under the Curve (AUCs) and cluster-robust standard errors. AI models were classified as Machine Learning Models (MLM) or Hybrid Nomograms (HN) based on their construction methodology.

**Results:**

Of 3,742 articles identified, 53 were included. MLMs demonstrated higher point estimates than radiologists in differential diagnosis (AUC: 0.87 vs. 0.83), though this difference was not statistically significant and carried substantial uncertainty. HNs achieved stronger performance in risk stratification (AUC: 0.87). AI-derived nomograms (AUC: 0.9) and gene signatures (AUC: 0.8) outperformed conventional prognostic markers descriptively. Chemotherapy response prediction remained below clinical utility thresholds across all model types. Only 33.9% of models reported calibration and 24.5% underwent external validation.

**Conclusions:**

AI demonstrates proof-of-concept across multiple NB clinical domains. However, clinical adoption remains premature given persistent gaps in external validation, calibration, dataset size, and pediatric-specific model development. Future studies should test these models prospectively in multicenter pediatric cohorts, ideally through COG or SIOPEN, using shared definitions for diagnosis, risk group, treatment response, and survival outcomes.

**Supplementary Information:**

The online version contains supplementary material available at https://doi.org/10.1007/s11912-026-01824-0.

## Key Results

- AI models mean performance match or exceed radiologist performance in neuroblastoma diagnosis.
- MLM outperform HNs in differential diagnosis.
- AI nomograms and gene signatures showed higher descriptive AUCs than several conventional prognostic markers, but formal comparative inference was not possible.
- Only 33.9% of models were calibrated; 24.5% underwent external validation.
- AI must transition from proof-of-concept to prospective clinical validation.

**Supplementary Information:**

The online version contains supplementary material available at https://doi.org/10.1007/s11912-026-01824-0.

## Introduction

Neuroblastoma (NB) is a rare and lethal pediatric cancer. Its prognosis is often poor, with an average 5-year overall survival (OS) of approximately 70%, although outcomes vary substantially according to risk classification [1] (1) NB is also characterized by marked biological and clinical heterogeneity, with disease courses ranging from spontaneous regression to rapidly progressive metastatic disease, contributing to significant pediatric cancer mortality [2].

Artificial Intelligence (AI) is increasingly being implemented into computer vision applications for both diagnosis and prognosis across various medical fields, particularly in ophthalmology and oncology [3, 4]. This process enhances the accuracy and efficiency of detecting diseases, predicting outcomes, and personalizing treatment plans [3, 4]. In prognosis, these new methods have already surpassed traditional evaluation techniques regarding early-stage lung cancer [5].

In imaging diagnosis, AI has increased accuracy, efficiency, and reproducibility across multiple modalities such as radiography, computed tomography (CT), magnetic resonance imaging (MRI), and digital pathology. Models, particularly convolutional neural networks (CNNs), automate the process and quickly process it. While doing so with matching or surpassing expert-level performance in tasks like cancer detection, fracture identification, and neurological disorder assessment, they also reduce inter-observer variability and clinical workload. Additionally, instead of replacing medical staff, large-scale studies and regulatory approvals increasingly support AI’s clinical utility, positioning it as a decision-support tool that augments clinical decisions, improves early diagnosis, and enables more personalized patient care [6, 7].

## Methods

This systematic review and meta-analysis was conducted following the PRISMA 2020 guidelines [8] and registered in PROSPERO registration (CRD42024539475).

Five electronic databases were searched on March 23, 2024: Embase, Scopus, PsycInfo, PubMed/MEDLINE, and Web of Science. Search commands were constructed according to other reviews [9, 10] and are provided in Supplementary_Material-Searches. Duplicate removal was performed using Mendeley, while title and abstract screening was conducted in Rayyan.

Studies were selected using the PICO framework [11]:

- Population: Human patients with NB, including diagnostic imaging datasets (CT, PET, MRI), digital pathology slides, and prognostic data.
- Intervention: Machine learning (ML) models applied to NB screening, prognosis, genomics, or pathology classification.
- Comparator: Any reference standard (neuroradiologist diagnosis, clinical follow-up, histopathology); comparators were not required.
- Outcome: Model discrimination (AUC preferred); sensitivity (SEN), specificity (SPE), accuracy (ACC), positive predictive value (PPV), negative predictive value (NPV), and hazard ratios (HRs) were also accepted.

Inclusion criteria: (1) studies involving human NB patients or representative datasets; (2) use of ML or ML-assisted techniques (including hybrid nomograms and gene signatures when ML was used for feature selection); (3) outcomes related to NB genetics, prognosis, or diagnosis; and (4) AUC or HR reported. Exclusion criteria: non-journal articles, reviews, and inaccessible full texts.

All stages of the systematic review were conducted independently by two reviewers (S.D. and L.A.) with a third reviewer (R. R.) independently resolving conflicts. Full-text decisions are documented in Supplementary_Material-Full-Reading-Decisions. Data extracted from each study included study design, sample size, dataset characteristics, machine learning algorithm, feature selection method and reported performance metrics.

Risk of bias occurred using a modified QUADAS-2 [12] tool that incorporated CONSORT-AI [13] criteria and additional AI-specific considerations [14] (Supplementary_Material-Modified-QUADAS-II). Domains evaluated included patient selection, index test, reference standard, and flow and timing.

Models were classified into two categories. Machine Learning Models (MLM) applied ML techniques in model construction (e.g., SVM, random forest, and neural networks). Hybrid Nomograms (HN) used ML only for feature selection, with the final prediction built as a conventional nomogram or signature. This distinction was used in all subgroup and meta-regression analyses.

### Statistical Analysis

Primary effect size: The AUC was used as the primary effect size because it provides a threshold-independent measure of discrimination and was the most consistently reported metric across included studies. A bivariate or hierarchical summary ROC approach was not feasible for the majority of studies, as individual contingency tables could not be reconstructed.

To make the statistical analysis, studies were subgrouped into different outcomes according to their objective. Outcomes with more than 3 articles were subject to meta-analysis.

As one study often reported multiple models that were included in the analysis, cluster-robust variance estimation was used using study ID as the clustering variable, with Satterthwaite-adjusted degrees of freedom to account for within-study dependence. AUCs were logit-transformed to stabilize variance and approximate normality, with standard errors derived from CI bounds.

Seven outcomes were analyzed: (1) risk stratification (RS); (2) differential diagnosis (DD) (NB vs. other diseases); (3) pathological classification, (4) Chemotherapy Response (CR); (5) MYCN amplification; (6) Bone Marrow Metastasis (BMM); and (7) risk factors (RF). Diagnostic performance across radiologists, HNs, and MLM was compared using separate meta-regressions for each parameter (SEN, SPE, ACC, RS AUC, and pathology AUC).

Pooled estimates were calculated overall and by model subgroups (ML and HNs). Differences between the two subgroups were assessed via meta-regression with model type as a categorical moderator, using the omnibus ***Q***-between statistic. Between-study heterogeneity was quantified using *tau*^2^, *I*^2^, and Cochran’s ***Q***. Reported AUC values and corresponding 95% CIs were logit-transformed, with standard errors calculated as:

$$\mathrm{SE}=\left(\mathrm{logit}\left(\mathrm{upper}\right)-\mathrm{logit}\left(\mathrm{lower}\right)\right)/\left(2\times1.96\right)$$

To account for within-study clustering, cluster-robust variance estimation was applied to the models. Results were then back-transformed to the AUC scale, and forest plots were generated showing individual and pooled estimates with heterogeneity metrics.

To compare pooled estimates with radiologists’ performance, a separate meta regression was used. The latter were specified as the reference category, and model type (Radiologist, HN, ML) was included as a categorical moderator. Given the limited number of effect sizes of radiologists (k = 3), fixed-effect meta-regression models with inverse-variance weighting were used, as random-effects estimation of τ² was not reliable. Coefficients represent log-odds differences relative to radiologists. The same process of moderator significance and logit transformation was applied to this analysis as well.

While moderator significance was evaluated using the omnibus QM statistic, individual coefficients used Wald tests. Pooled estimates for each model were obtained by combining the intercept and moderator coefficients on the logit scale and back-transforming via the inverse logit function. Residual heterogeneity was reported (QE, I², H²), although it should be interpreted cautiously due to the small sample size of radiologists.

Outcomes that had only one independent study cluster were not considered eligible for random-effects meta-analysis or cluster-robust inference. When such outcomes included multiple AI models reported, descriptive means were calculated through an inverse-variance weighted random-effect summary. These means were used as proxies for comparison with radiologist performance and were not interpreted as formal pooled meta-analytic estimates. No between-study heterogeneity, prediction intervals, or subgroup-difference tests were reported for these cases.

### Secondary Parameters

Prognostic and genetic outcomes: HRs were pooled using a generic inverse-variance approach (metagen function, meta package in R). Studies were grouped by model type (MLM vs. HN) via subgroup analysis. Between-study heterogeneity was assessed with I² and τ². For outcomes where 95% CIs were not available, unweighted arithmetic means were calculated within subgroups for descriptive comparison only. This descriptive analysis was used for secondary parameters (ACC, SEN, SPE, PPV, NPV), prognosis AUC’s markers synthesis, temporal analysis of prognosis´AUC.

All analyses were performed in R using the metafor and clubSandwich packages. Meta-analyses were conducted only when three or more eligible studies reported the relevant outcome.

Finally, large language models (ChatGPT 5.5 and Claude Sonnet 4.6) were used to assist with language editing and adaptation of R scripts used in the statistical analysis. All final versions of analytical scripts, and manuscript writing were reviewed and verified by the authors.

## Results

### Characterization of Studies

**Fig. 1:**
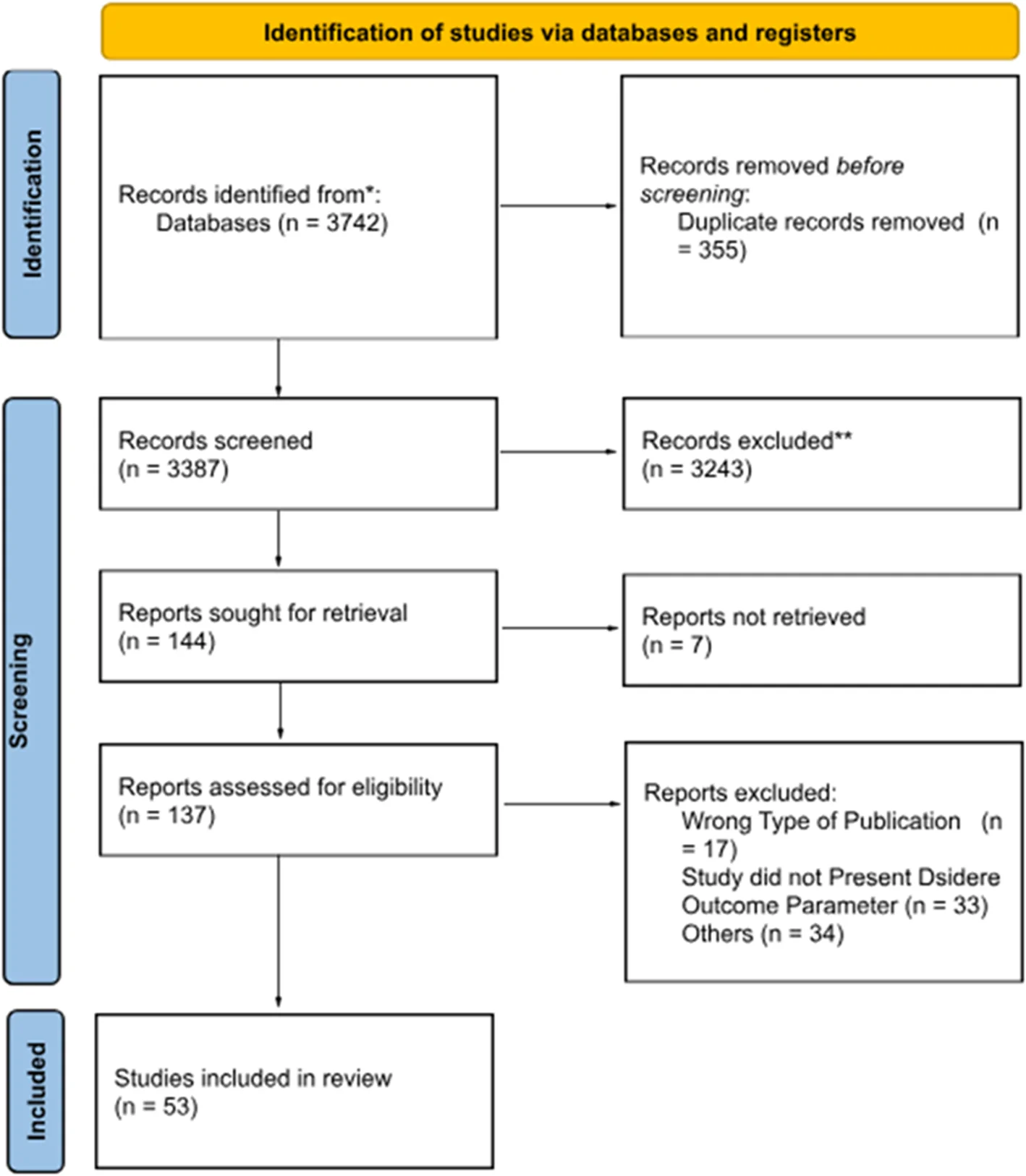
- Fluxogram of the screening process

The initial search yielded 3,742 articles. After removing 355 duplicates, 3,387 records were screened for eligibility. Of these, 3,233 were excluded for not meeting the inclusion criteria, leaving 137 studies for full-text assessment. Following full-text review, 84 articles were further excluded, resulting in a final sample of 53 studies included in this review. The rationale of full article reading can be found in Supplementary_Material-Full-Reading-Decision. Figure 1 shows this process.

The 53 included studies were divided according to their outcome: RF (15, 28.30%) [15–28], prognosis (14, 26.42%) [15, 18–22, 24–26, 28–32], MYCN amplification (9, 16.98%) [30, 33–40], genes (8, 15.09%) [15, 22, 27, 28, 41–43]RS (6, 11.32%) [36, 44–48], DD (5, 9.43%) [49–53], BMM (5, 9.43%) [30, 54–57], pathology (4, 7.55%) [58–61], CR (3, 5.66%) [62–64], early and late relapse (2, 3.77%) [43, 65], and recurrence (1, 1.89%) [66]. Some articles were classified into multiple categories as their results involve more than one type of data. Their full characteristics can be seen in Supplementary_Material-Studies-Characterization.

**Fig. 2:**
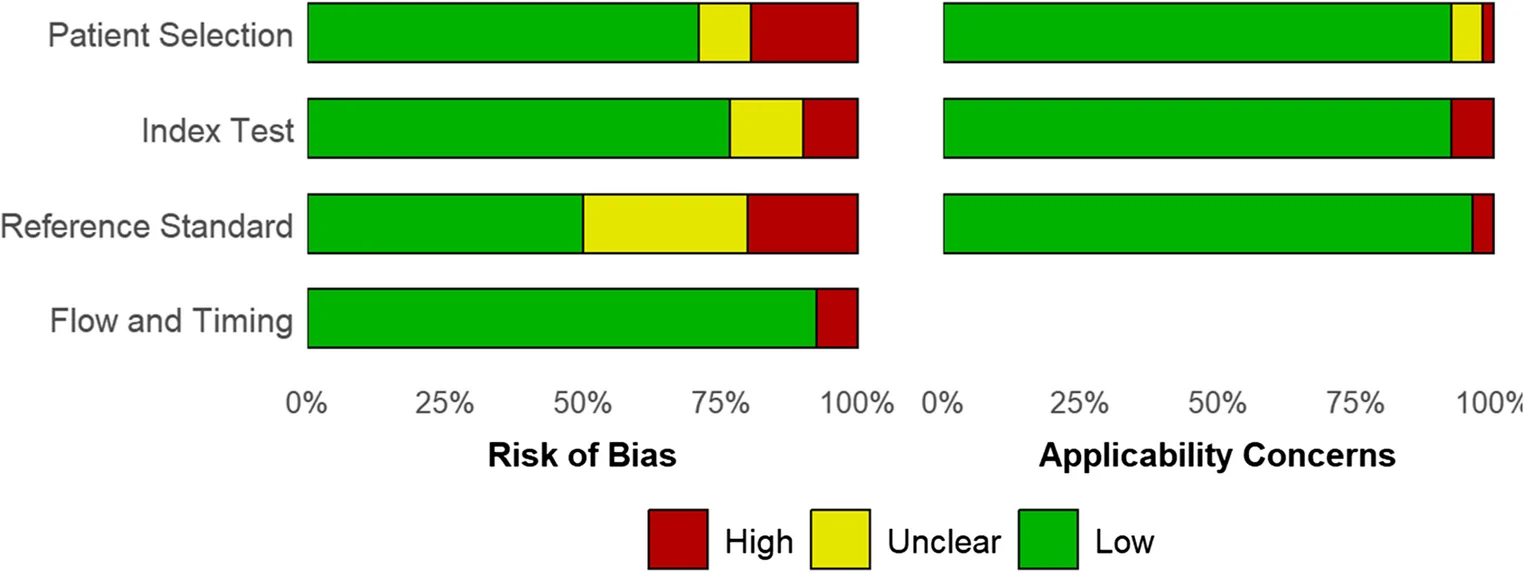
- Risk of bias description. Scores were obtained through the assessment using the modified tool QUADAS-II

Figure 2 demonstrates the results of the QUADAS-II risk of bias assessment across patient selection (PS) and index test (IT), reference standard and flow and timing domains. PS and IT domains demonstrated the highest methodological rigor, with 92.5% (*n* = 49) and 77.4% (*n* = 41) of studies classified as low risk, respectively. Conversely, the reference standard domain exhibited the greatest degree of uncertainty and potential bias; only 50.9% (*n* = 27) of studies were rated as low risk, while 30.2% (*n* = 16) were classified as unclear and 20.8% (*n* = 11) as high risk.

In the initial PS category, 71.7% (*n* = 38) of the studies were found to have a low risk of bias, while 18.9% (*n* = 10) and 9.4% (*n* = 5) were categorized as high and unclear risk, respectively. Notably, the IT domain showed a minimal high-risk profile (9.4%, *n* = 5), though 13.2% (*n* = 7) of studies remained unclear. Overall, the second PS domain showed the most definitive results, with no studies (0%) falling into the unclear category.

### Diagnosis

Table 1 presents the AUC values for each evaluated outcome across the three compared methods (HN, MLM, and Radiologist). For DD, AUC values were 0.82 (95% CI: 0.8–0.84), 0.87 (95% CI: 0.18–0.99), and 0.83 (95% CI: 0.73–0.93, *n* = 2) for HN, MLM, and Radiologist, respectively. For RS, AUC values were 0.87 (95% CI: 0–1), 0.81 (95% CI: 0.07–0.99), and 0.724 (95% CI: 0.569–0.848, *n* = 2) for HN, MLM, and Radiologist, respectively. 

Pathology classification’s articles only had MLM, whose AUC’s was 0.82 (95% CI: 0.71–0.89). For CR prediction, HN and MLM achieved AUCs of 0.77 (95% CI: 0.70–0.83) and 0.67 (95% CI: 0.61–0.73), respectively. MYCN status prediction yielded AUCs of 0.85 (95% CI: 0.57–0.96) and 0.75 (95% CI: 0.49–0.9) for HN and MLM, respectively. Finally, for BMM prediction, AUC values were 0.8 (95% CI: 0.67–0.89) and 0.8 (95% CI: 0.15–0.99) for HN and MLM, respectively. The forest plots of every outcome can be found in Supplementary_Material-Forest-Plots.

**Table 1:** Pooled area under the curve of Hybrid Nomograms (HN), Machine Learning Models (MLM) and Radiologists across different outcomes

Outcome	HN	MLM	Radiologists
Differential Diagnosis	0.82 (0.8–0.84)	*0.87 (0.18–0.99)	0.83 (0.73–0.93, *n* = 2)
Risk Stratification	*0.87 (0–1)	*0.81 (0.07–0.99)	0.72 (0.57–0.85, *n* = 2)
Pathology	-	0.82 (0.71–0.89)	-
Chemotherapy Response	0.77 (0.70–0.83)	0.67 (0.61–0.73)	-
MYCN amplification	0.85 (0.57–0.96)	0.75 (0.49–0.90)	-
Bone Marrow Metastasis	0.8 (0.67–0.89)	*0.8 (0.15–0.99)	-

* CI width > 0.5 AUC units; pooled estimate reflects insufficient data for precise inference and should be interpreted descriptively

### Comparison Between MLM, HN and Radiologists

Radiologists’ AUC performance was calculated to be: 0.83 (0.73–0.93) (*n* = 2) [67] for DD and 0.72 (0.57–0.85) (*n* = 2) [68] for RS. The mixed-effects meta-regression (Radiologist, HN, or MLM) and REML estimation (k = 6) yielded low and not statistically significant residual heterogeneity (τ² = 0.039; I² = 8.14%; QE(3) = 1.29, *p* = 0.73).

The test of moderators demonstrated that model type did not significantly influence AUC performance (QM(2) = 0.82, *p* = 0.664). On the logit scale, neither HN (β = 0.34, SE = 0.37, *p* = 0.37) nor MLM (β = 0.44, SE = 2.88, *p* = 0.88) differed significantly from radiologist´s performance.

### Secondary Parameters

For DD, the MLM outperformed the HN in ACC (0.92 vs. 0.82) and SPE (0.97 vs. 0.77), though the HN showed higher SEN (0.78 vs. 0.73). Both models demonstrated strong PPV and NPV, with the MLM slightly ahead (PPV: 0.93 vs. 0.85; NPV: 0.87 vs. 0.8). In RS, the HN achieved marginally better ACC (0.82 vs. 0.8) and SEN (0.82 vs. 0.75), while the MLM had higher SPE (0.88 vs. 0.79). PPV and NPV were not available (-) for this outcome.

HN that predicted CR showcased an AUC, SEN, SPE, ACC, PPV and NPV, respectively, of: 0.77 (0.70–0.83), 0.7, 0.9, 0.83, 0.82 and 0.83. MLM showcased only an AUC of 0.67 (0.61–0.73). HN and MLM that predicted BMM occurrence presented an AUC, respectively, of 0.80 (0.67–0.89) and 0.80 (0.15–0.99).

For MYCN Amplification, the models performed similarly in ACC (HN: 0.76; MLM: 0.75), with the MLM showing slightly higher SEN(0.76 vs. 0.728) and the HN exhibiting better SPE (0.858 vs. 0.78). Notably, the MLM achieved a perfect PPV of 1, while the HN’s PPV was not reported in articles. In Pathology, only the MLM was evaluated, with moderate performance across all metrics (ACC: 0.8; SEN: 0.81; SPE: 0.8; PPV: 0.87; NPV: 0.74). For BMM, the HN’s performance was weaker (ACC: 0.62; SEN: 0.62; SPE: 0.6), and MLM data were unavailable. As shown in Table 2.

**Table 2:** Secondary parameter of artificial intelligence models per outcome

Outcome	Parameters									
	Accuracy		Sensitivity		Specificity		PPV		NPV	
	HN	MLM	HN	MLM	HN	MLM	HN	MLM	HN	MLM
Differential Diagnosis	0.82	0.92	0.78	0.73	0.77	0.97	0.85	0.93	0.8	0.87
Risk Stratification	0.82	0.8	0.82	0.75	0.79	0.88	-	-	-	-
MYCN amplification	0.72 (0.57–0.84)	0.71 (0.58–0.81)	0.728	0.76**	0.858	0.78**	-	1***	-	0.83
Pathology	-	0.8	-	0.81	-	0.8	-	0.87	-	-
Bone Marrow Metastasis	0.62 (0.53–0.70)*	-	0.65 (0.5–0.77)*	-	0.6 (0.49–0.7)*	-	-	-	-	-

*HN* Hybrid Nomogram, *MLM* Machine Learning Model, *AUC* Area Under the Curve

* Data from External Validation

** Two outliers models were removed

***Data from only 1 model

### Prognosis

Table 3 presents the mean AUC values of AI-based prognostic models and conventional clinical factors across three studies [15, 21, 22], which in total encompasses 4,318 patients.

Among evaluated markers, AI-derived ones showed higher reported AUC values compared with conventional clinical markers. Nomograms achieved the highest mean AUC (0.901, derived from 4 estimates across a single study), followed closely by gene signatures (0.801, 4 estimates). These values represent unweighted arithmetic means calculated in the absence of extractable confidence intervals and should be interpreted as descriptive rather than inferential.

Conventional clinical factors´ performance was reported as: Age at diagnosis yielded a mean AUC of 0.788 (5 estimates, 2 studies), risk scores 0.816 (1 estimate), and MYCN stage 0.762 (5 estimates, 3 studies). COG Risk Group achieved a mean AUC of 0.730 (2 estimates, 2 studies). Several conventional factors showed relatively low AUC values in the available studies: MKI (0.589), Gender/Sex (0.514), INSS Stage (0.472), Grade (0.425), and Ploidy Value (0.303), all derived from a single study [15].

Vital Status (0.896) and Stage 4 vs. 4 S (0.888) demonstrated high AUC values, though both estimates derive from a single study [22] with a single reported value each, precluding meaningful averaging and limiting interpretability.

**Table 3:** Descriptive summary comparison between Artificial Intelligence signatures, nomograms and other clinical factors in predicting overall survival. Standard deviations (SD) are reported to illustrate the variability of AUC values across the available model estimates rather than to represent pooled statistical uncertainty

Variable	Mean AUC and SD	Number of patients/estimates
Nomogram	0.901 (0.09)	1641/4 [21]
Vital Status	0.896	561/1 [22]
Stage 4 vs. 4 S	0.888	561/1 [22]
Risk Score	0.816	2116/1 [15]
Signature	0.801 (0.17)	1641/4 [21]
Age	0.788 (0.15)	3757/5 [15, 21]
MYCN Stage	0.762 (0.2)	4318/5 [15, 21, 22]
COG Risk Group	0.73 (0.19)	3757/2 [15, 21]
MKI	0.589	2116/1 [15]
Gender/Sex	0.514 (0.05)	3757/3 [15, 21]
INSS Stage	0.472	2116/1 [15]
Grade	0.425	2116/1 [15]
Ploidy Value	0.303	2116/1 [15]

**Fig. 3:**
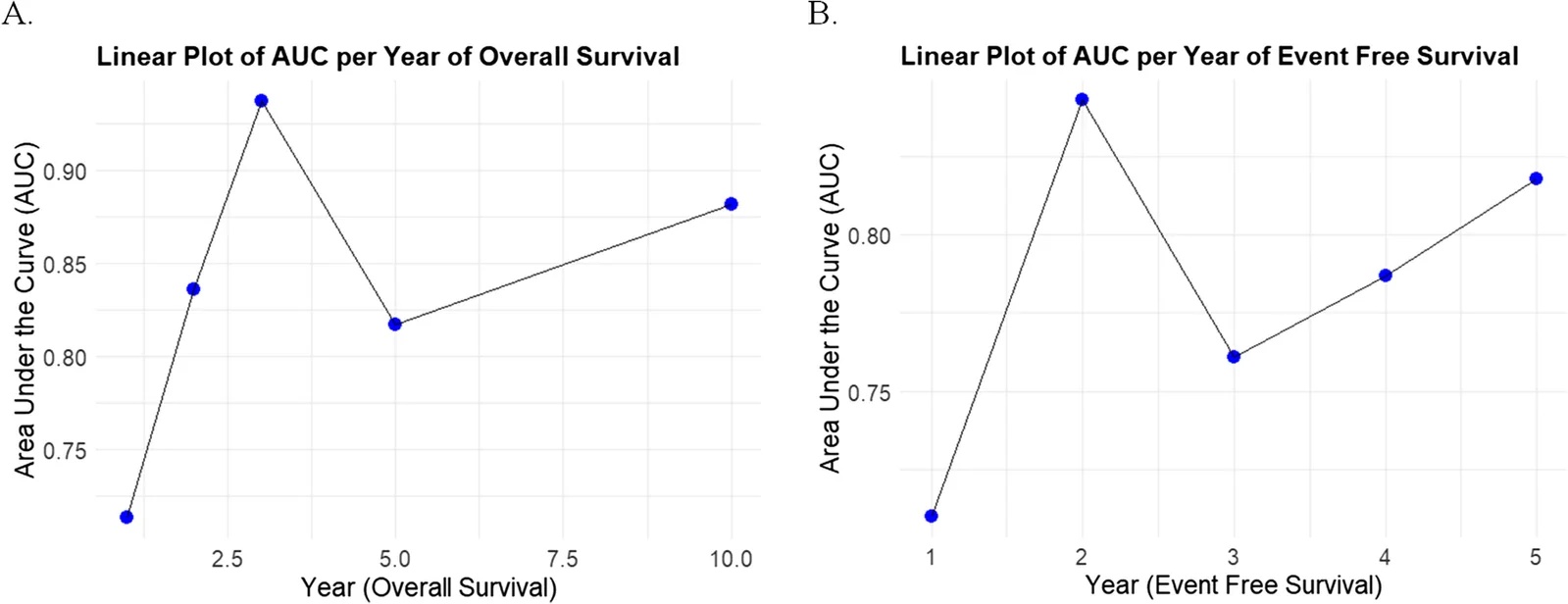
- Performance (in AUC) of models in predicting prognosis, (**A**) overall survival and (**B**) Event Free Survival, of neuroblastoma patients

The temporal analysis of model performance’s results is presented in Fig. 3. For overall survival (OS), 7 models across 5 studies [18–21] predicted outcomes at 1, 3, and 5 years, encompassing 6,246 patients, while a single study [18] provided a 10-year estimate based on 721 patients. No 2-year or 4-year OS estimates were available. For event-free survival (EFS), 7 models across 3 studies [24–26] predicted 1-year outcomes in 458 patients, and 4 models across 2 studies [24, 25] predicted 2-year outcomes in 286 patients. Beyond 2 years, EFS estimates were limited to a single study and single model [24](167 patients) at each of the 3-, 4-, and 5-year time points.

Across both variables, model reported AUC values appeared lower for 1-year predictions, a peak in 2-year for EFS and a 3 year for OS. A sharp drop and an increasing trend with year is observed after the peak in both variables. However we highlight the descriptive nature of the prognosis analysis.

Furthermore, several studies also reported HRs for prognostic models and clinical variables. Gene expression signatures predicting OS reported HR values below 2.31 in multivariate Cox analyses [22, 28]. In comparison, some AI-derived models reported higher HR values, including an IGPM-based risk signature [15] (HR 2.743) and an artificial neural network model [18] (HR 5.687). However, conventional clinical predictors in some studies demonstrated even higher HR values, such as COG risk group [15] (HR 4.799) and INSS stage 4 [23] (HR 11.091).

Age as a whole did not demonstrate a HR higher than NG, however subdivision of it do demonstrate a higher HR: cutoffs at 18 months provided an HR of 3.1 to those above it and 6.12 to those below [20]; In univariate COX, at 9 months demonstrated a HR of 2.3 (1.1–4.9), 6 months, 2.1 (0.7–6.0) and 3 months, 8.9 (0.2–3000) [17]. Only a Rad Score based signature [24] presented a HR of 4.588 (2.278, 9.243).

### Genes

The main upregulated genes considered candidates (*p* < 0.05) by AI models for NB were GALGT/SLC26A10, AIPL1, ULK2, GUCY2F, DLG3, RIN2, UGT2A1, RAB3GAP, GPC1, TNK2 [43], FARSA, SLC29A2, PLK1, WDR74, RRP9, and IMP4 [22], while the downregulated genes were CMTR1, NUB1, and STAT2 [22], DCX, MYH10, BCL11A, EGFL4, LRRC48, NPPA, KCNJ6, PCSK1, PDE6C, NTSR1 [43]. Other genes related to the immune system and considered candidates were: SOCS1, MARCO, KLRK1, IRF7, UNC93B1, IGHV3–20, IGKV1–16, AMH, SECTM1 [15]. Two genes encoding plexin receptors, PLXNA4 and PLXNC1, were also considered candidates [28].

### MYCN

In a radiogenomics prediction of MYCN amplification through imaging exams, HN and MLM obtained AUC, SEN and specificities of, respectively: 0.85 (0.57–0.96) and 0.75 (0.49–0.90); 0.728 and 0.58; 0.858 and 0.847. However if 2 MLM outliers models (< 0.2) are removed, then this classification presents an SEN and SPE of, respectively 0.76 and 0.784.

## Discussion

### Clinical Implications

As no predetermined classification of ranges of ACC, SEN, SPE exist [69], an AUC threshold of 0.8 can be adopted to be considered clinically useful [70]. From the interpretation of results, the only median AUC which was lower than the threshold was the “Reached the criterion” outcome, however it should also be noted that while BMM has an median AUC of 0.8, their ACC, SEN and SPE were less than ideal (< 0.8).

This meta-analysis demonstrates that AI models, particularly MLM and HN, show important promise in improving NB diagnosis, RS, and prognostic assessment. However, important implications must be taken into account before implementation in pediatric oncology practice.

MLM demonstrated a higher point estimate for DD AUC compared to radiologists (0.87 vs. 0.83), though this difference was not statistically significant and the pooled MLM estimate carried substantial uncertainty (95% CI: 0.18–1.00), due to the small number of contributing studies.

AI-based nomograms and gene signatures demonstrated higher mean AUC values (0.901 and 0.801, respectively) than conventional prognostic factors, including age at diagnosis (0.788), risk scores (0.82), and MYCN stage (0.76). These comparisons are based on unweighted arithmetic means calculated in the absence of extractable confidence intervals and should be interpreted as descriptive rather than inferential, no formal test of superiority was conducted. Nonetheless, the consistent directional trend across multiple AI model types and conventional factors suggests that AI-assisted prognostic tools warrant further prospective investigation as potential complements to existing risk stratification frameworks.

Despite point estimates suggesting that AI models match or exceed radiologist performance in certain outcomes, several barriers must be addressed before widespread clinical adoption can be recommended. For reasons such as (1) Lack of generalizability and external validation, which when present tend to lower models’ performance [71–73]; (2) Integration and workflow challenges, due to lack interoperability, transparency, and user-friendly interfaces, the models implementation do not Always occur.smoothly. And, in presence of errors, patient’s trust is often eroded, resulting in a drop of service’s quality and elevation of costs [72, 73]; (3) Insufficient prospective and real-world clinical evaluations, with few prospective studies or randomized controlled trials assessing their impact on clinical outcomes or workflow integration [74–76].

Prognosis temporal analysis, despite its descriptive nature, indicates interesting trends, such as the models´ best performance in moderate times (2–3 years) sandwiched by worse performance (1 year and 4–5 years). This could be due to the critical risk stratification horizon, which separates patients who achieved remission from those with relapses, another important factor is that the training set may have been optimized for this timeframe, as indicated by the fact that no 2 year or 4 year models were present in the OS variable. Further aspects that can inhibit post 4–5 year predictions: survivorship bias [77], heterogenous biological aspects in late cases [78, 79], loss of follow up [79, 80]. And ones that can contribute to underperformance in 1 year: censoring bias [81], competing events creating complex patterns [82], prevalence of heterogeneous aggressive fatal NB [83].

A promising triage tool characterized by the ability of AI models to predict MYCN amplification non-invasively through imaging analysis (AUC 0.85 for HN, 0.75 for MLM) is better discussed in another meta analysis encompassing only this theme [84].

However, one outcome that demonstrates unrealized potential is CR, which demonstrated an AUC of MLM and HN of 0.67 (0.61–0.73) and 0.77 (0.70–0.83) respectively, noticeably lower than other outcomes. NB is a deadly, heterogeneous and rapidly progressing disease, therefore having a non-invasive tool capable of predicting response to treatment would be of great use. However, models performance is currently only a little bit better than coin flipping’s chance (AUC = 0.5).

This phenomenon could be happening for a few reasons: Tumor heterogeneity, NB tumors are biologically diverse even within the same risk group. Different clones within a single tumor may respond differently to treatment, making any single imaging or genomic snapshot an incomplete picture; Treatment protocol complexity, NB chemotherapy regimens are multidrug and adaptive. The “response” being predicted is itself a moving target depending on which cycle, which drugs, and which response criteria are used; Definition inconsistency, studies likely define “response” differently (RECIST, INRG criteria, scintigraphy response), making pooling inherently noisy; and ultimately and arguably most important a small number of studies, only 3 studies contributed to this outcome. And when this small number is combined with the other cited reasons, data produced may be too heterogeneous for MLM to find a pattern.

### Pediatric Specific Problems

One aspect encountered specifically on NB studies is that of data availability. Children’s imaging data are less abundant and more heterogeneous than adult datasets, which leads to challenges in training robust AI models and increasing the risk of overfitting or poor generalizability to diverse pediatric populations [85–87]. Another aspect of difficulty in AI training is physiological and anatomical differences across pediatric age groups, as algorithms trained on adults or older children may not perform accurately in younger or developmentally distinct patients [88, 89]. Several societies, ACR, ESPR, SPR, SLARP, AOSPR, and SPIN, recognize this problem and released a multi-society statement specifically emphasizing the need for pediatric-specific safety ratings, inclusion of diverse pediatric datasets, and continuous post-market surveillance to ensure safety and efficacy in children [88].

Bias is a significant concern, as underrepresentation of certain pediatric subpopulations (e.g., neonates, rare diseases, or specific ethnic groups) can lead to inequitable performance and potential harm [90, 91]. Additionally, most commercially available AI tools are developed and validated in adult populations, and their direct application to pediatric imaging can result in suboptimal diagnostic ACC unless thresholds and models are specifically adapted and validated for children [89]. Finally, ethical and regulatory challenges, including transparency, explainability, and the need for ongoing education of pediatric radiologists and clinicians, are critical to ensure safe and effective AI integration in pediatric care [85, 88, 90].

### Calibration and External Validation

One crucial aspect that many models did not possess is calibration. It can be defined as the ACC of risk estimates, relating to the agreement between the estimated and observed number of events and is an important aspect of diagnosing model. Poor calibration can make predictions misleading, further explanation of this topic is referenced to [92, 93].

Only 18 (33.9%) of models were calibrated, 2 out of 6 (33%) for RS, none out of DD, 4 out of 9 (44%) for MYCN amplification, 10 out of 13 (77%) for prognosis, 2 out of 3 (66%) for pathology.

Only 13 (24.5%) presented external validation of their models, 1 which measured segmentation [50] and presented good results - an AUC of 0.93 (+- 0.191), SEN, 0.847 (+- 1.123), SPE, 0.917 (+- 0.215) - and one which predicts bone marrows involvement [56] and presented mixed results - all 4 NM had AUC, SEN, SPE and ACC lower than 0.8. Additionally, only the combined NM didn’t showcase a large drop of ACC between internal and external validation.

In RS [48], 2 out of 3 models showcased an AUC higher than 0.8 and 1 ACC higher than this threshold. In DD [49], 4 models showcased AUCs higher or equal to 0.8 however its ACC were lower than 0.8. In MYCN amplification [38], 3 models had an AUC lower than 0.8 and 2 had SPEs of 1, but SENs of 0.1429. In pathology [60], 4 models achieved an AUC higher or equal to 0.9 and 3 models, an ACC higher than 0.8.

A 2023 systematic review [94] about the differences between internal and external AUC performance, found that, on average, AUC was reduced by −0.037 (95% CI −0.052 to −0.027). Out of an ongoing systematic review in DD of thymomas, out of 27 models, 1 showcased an AUC lower than 0.8 and 7 had an ACC lower than 0.8 [95, 96].

Due to lacking numbers, the same cannot be said in this review. Therefore, rigorous internal validation is highlighted and recommended, as failed external validation can be foreseen by it [97].

### Technical Aspects

The included studies applied a wide range of machine learning algorithms, including logistic regression, linear discriminant analysis, support vector machines, random forests, gradient boosting methods, and neural networks. Across several studies, simpler linear models performed worse when compared to more complex nonlinear algorithms, particularly when dataset sizes were limited [45, 60]. This finding suggests that model complexity alone does not guarantee improved predictive performance in NB datasets, which are often relatively small. Several studies also reported evidence of overfitting, reflected by substantial differences between training and validation AUC values [36, 52]. In addition, many articles did not clearly describe hyperparameter optimization procedures or search spaces, making it difficult to reproduce model performance [49]. Improved reporting of model development pipelines, including feature selection strategies and hyperparameter tuning procedures are important for enhancing reproducibility and comparability in future studies.

Beyond the calibration steps mentioned above, several additional best practices are worth highlighting. First, all hyperparameters should be clearly reported to ensure reproducibility, including those of the machine learning algorithms and any applied to data preprocessing. Second, researchers should explore multiple hyperparameter configurations, documenting the search space and optimization strategy used [98]. Overfitting must be carefully considered, particularly when working with small datasets; strategies such as leave-one-out cross-validation can help produce more reliable performance estimates.

### Limitations

This systematic review and meta-analysis has several important limitations that should be considered when interpreting the findings. (1) The majority of included studies were retrospective in design. (2) External validation was performed in only 24.5% of included studies, and calibration was assessed in only 33.9% of models. (3) Significant heterogeneity existed across included studies in terms of patient populations, imaging protocols, AI architectures, feature selection methods, and outcome definitions. (4) Many studies did not clearly describe consecutive enrollment or blinding of outcome assessors, potentially introducing bias. (5) The analysis of model performance across different time periods for prognosis prediction was limited by the small number of models available for each specific time point, particularly beyond three years.

### Future Directions

#### Existing Clinical Networks for Multicenter Validation

One solution to NB’s rarity is collaboration between institutions, whose infrastructure already exists. The Children’s Oncology Group (COG) and the International Society of Paediatric Oncology Europe NB group (SIOPEN) coordinate multinational clinical trials and maintain longitudinal patient datasets with standardized staging, treatment protocols, and outcome definitions. Future research should prioritize embedding AI model development and validation directly within these existing networks, rather than building parallel data-sharing mechanisms from scratch. Prospective collection of imaging, genomic, and clinical response data within ongoing COG and SIOPEN trials would provide the scale and standardization that retrospective single-institution studies cannot achieve.

#### Federated Learning

Another method to overcome a small N is federated learning. Although some barriers exist such as heightened privacy concerns in pediatric populations, and cross-national data sharing faces significant legal and ethical complexity across SIOPEN member countries and between COG institutions. Additionally, heterogeneity in imaging protocols, scanner manufacturers, acquisition parameters, and annotation practices across institutions can introduce systematic bias that federated training does not automatically resolve. Future studies adopting federated architectures should prospectively address harmonization of imaging protocols, standardization of labeling procedures, and validation of model performance across institutional subgroups to ensure that federated models generalize equitably rather than performing well on average while failing in specific subpopulations.

#### Dedicated Pediatric Neuroblastoma Data Infrastructure

Adult oncology AI has benefited substantially from large, publicly accessible datasets such as The Cancer Imaging Archive. Pediatric equivalents are sparse, and NB-specific public datasets are practically nonexistent. Consequently, each new research group begins from scratch with small institutional cohorts, perpetuating the very limitations, small sample sizes, single-center design, lack of external validation, that this review identifies. We recommend the creation of a curated, publicly accessible NB imaging and genomics repository, developed collaboratively through COG and SIOPEN, with standardized image acquisition protocols, unified annotation frameworks, and consensus-defined clinical endpoints.

#### Prioritizing Chemotherapy Response Prediction

Among all outcomes examined in this review, CR prediction represents both the greatest current gap and the highest potential clinical payoff. Current models fall below the accepted clinical utility threshold (AUC < 0.8), and only three studies contributed to this outcome. Yet its clinical potential is enormous: such as supporting de-escalation strategies for patients demonstrating strong early responses or identifying patients resistant to chemotherapy.

Advancing this outcome will require addressing two foundational problems before further model development. First, a standard definition of chemotherapy response must be established across institutions, current studies use heterogeneous criteria (RECIST, INRG response criteria, scintigraphy-based assessment) that make pooling noisy and cross-study comparison unreliable. Second, baseline-only imaging models are likely insufficient for this task given the dynamic nature of treatment response. Longitudinal multimodal models that incorporate serial imaging across treatment cycles, integrated with genomic and clinical biomarker trajectories, represent the most promising architectural direction. Recurrent neural networks and transformer-based architectures specifically designed for temporal data sequences warrant prioritized investigation in this context.

#### Development of Interpretable Hybrid Architectures

This review identified a consistent pattern. MLMs demonstrated higher point estimates in DD, while HNs outperformed MLMs in RS. This finding poses a question that the current literature has not adequately addressed: should future development favor one architecture, or can the complementary strengths of each be combined? It is suggested that hybrid architectures, models that apply ML techniques to both feature selection and final prediction while constraining outputs to interpretable, clinician-readable formats, represent a productive direction.

#### Optimizing Prognostic Models Across the Full Temporal Spectrum

The temporal analysis presented in this review reveals a consistent pattern of underperformance at the 1-year and post-4-year time points, with peak model performance concentrated at 2–3 years. These observations are not merely statistical in nature; rather, they reflect identifiable biological and methodological phenomena that must be directly addressed in the development of future models.

In order to address the issues that have been previously examined on an annual basis. At the one-year mark, the incorporation of competing risk methodologies that explicitly account for early treatment-related mortality alongside disease progression is imperative in future models. Furthermore, the development and validation of models should be conducted on long-term survivor cohorts, distinct from early-phase models and patients, extending beyond a four-year period. Furthermore, longitudinal architectures incorporating serial imaging and biomarker data collected throughout the post-treatment surveillance period, rather than relying on baseline snapshots alone, offer the most conceptually appropriate framework for late prognostic prediction.

#### Navigating the Pediatric Regulatory and Ethical Landscape

The regulatory pathway for AI tools in pediatric oncology is distinct from adult applications and is currently underdeveloped relative to clinical need. The FDA’s predetermined change control plans and the EU AI Act’s high-risk classification for medical AI both carry specific implications for pediatric applications that the NB AI community has not yet systematically engaged with. The multi-society statement issued by ACR, ESPR, SPR, SLARP, AOSPR, and SPIN, cited in this review as evidence of a recognized problem, should be treated as a practical framework for action: its recommendations for pediatric-specific safety ratings, inclusion of diverse pediatric datasets, and continuous post-market surveillance should be operationalized as requirements in future study design, not aspirational guidelines.

In addition to ensuring regulatory compliance, it is imperative to acknowledge the ethical obligations that are unique to pediatric AI. The underrepresentation of neonates, infants, and specific ethnic groups in training datasets can result in the development of models that demonstrate inequitable performance across subpopulations that are particularly in need of enhanced diagnostic tools. The establishment of age-specific model calibration and stratified validation across developmental subgroups should be considered non-negotiable standards rather than aspirational goals. It is imperative that continuous post-deployment monitoring protocols be incorporated into AI implementation plans from the outset. These protocols are designed to detect performance degradation as imaging technologies, treatment protocols, and patient demographics evolve.

## Conclusion

The evidence produced in this review permit the affirmation that AI-based models can match experts’ in certain domains. Yet, the vast majority of them are not validated, only 33.9% of models were calibrated and only 24.5% underwent external validation. Additionally, certain outcomes, specially CR, remain below the threshold for clinical utility.

NB presents specific structural challenges that generic AI development frameworks do not address: extreme data scarcity, pediatric physiological heterogeneity across developmental stages, heightened privacy and ethical obligations, and a regulatory landscape that has not kept pace with technological capability. These are not problems that more algorithms will solve. They require coordinated action, through COG and SIOPEN networks, through dedicated pediatric data infrastructure, through consensus outcome definitions, and through regulatory frameworks designed specifically for children. Before clinical implementation, the field needs fewer single-center proof-of-concept models and more externally validated, calibrated, prospectively tested tools.

## Supplementary Information

Supplementary Material 1.

Supplementary Material 2.

Supplementary Material 3.

Supplementary Material 4.

Supplementary Material 5.

## Data Availability

Data utilized in this article is available through Supplementary Material files.

## Key References

- Shelmerdine SC, Naidoo J, Kelly BS, Laborie LB, Toso S, Akinci D’Antonoli T, et al. AI implementation in pediatric radiology for patient safety: a multi-society statement from the ACR, ESPR, SPR, SLARP, AOSPR, SPIN. Pediatr Radiol. 2026 Feb 1;56(2):243–56. https://doi.org/10.1007/s00247-025-06386-0
  - The is the most authoritative voice on the pediatric-specific challenge, for those who want to read more about specific recommendations introduced in this review are referred to this statement.
- Wang H, Ji Y, Chen X, He L, Fang X, Cai J. Radiomics-Based Machine Learning for Determining MYCN Amplification Status in Childhood Neuroblastoma: A Systematic Review and Meta-Analysis. Technol Cancer Res Treat. 2025;24:15330338251358324. https://doi.org/10.1177/15330338251358324. PubMed PMID: 40619885; PubMed Central PMCID: PMC12235222.
  - This systematic review and meta analysis goes into detail about the application of AI into MYCN non-invasive triage through image analysis. Which complements our MYCN findings directly, providing deeper methodological detail on radiomics-based non-invasive prediction.
- Xia Y, Wang C, Li X, Gao M, Hogg HDJ, Tunthanathip T, et al. Development and validation of a novel stemness-related prognostic model for neuroblastoma using integrated machine learning and bioinformatics analyses. Transl Pediatr. 2024 Jan 29;13(1):91–109. https://doi.org/10.21037/tp-23-582. PubMed PMID: 38323183; PubMed Central PMCID: PMC10839279.
  - Among the included studies, this is one of the most methodologically complete in the prognosis outcome, currently the most underdeveloped. It reports both internal and external validation with a clinically meaningful prognostic model. For readers who want to see an example of validation, biological grounded AI models in NB.
