# Supplementary material for: Artificial Intelligence for Diagnosis, Risk Stratification, and Prognosis of Neuroblastoma - A Systematic Review and Meta-Analysis
Source: Curr Oncol Rep. 2026 Sep 4;28(1):90. doi: 10.1007/s11912-026-01824-0 (PMC13545110; doi:10.1007/s11912-026-01824-0)
Supplement: Supplementary file 1 [file 11912_2026_1824_MOESM1_ESM.docx]

**Databases Searches**

**PubMED**

((Radiomics) OR (Computational Intelligence) OR (Intelligence, Computational) OR (Machine Intelligence) OR (Intelligence, Machine) OR (Computer Reasoning) OR (Reasoning, Computer) OR (AI (Artificial Intelligence)) OR (Computer Vision Systems) OR (Computer Vision System) OR (System, Computer Vision) OR (Systems, Computer Vision) OR (Vision System, Computer) OR (Learning, Machine) OR (Machine Learning) OR (Transfer Learning) OR (Learning, Transfer) OR (Vision Systems, Computer) OR (Knowledge Acquisition (Computer)) OR (Acquisition, Knowledge (Computer)) OR (Knowledge Representation (Computer)) OR (Knowledge Representations (Computer)) OR (Representation, Knowledge (Computer))) AND (Neuroblastoma)

(AI OR AI1 OR AI2 OR AI3 OR Radiomics) AND ((NB) OR “NB1” OR (NB2) OR NB3)

**Embase**

Radiomics OR Learning, Machine OR Machine Learning OR Transfer Learning OR Learning, Transfer OR Computational Intelligence OR Intelligence, Computational OR Machine Intelligence OR Intelligence, Machine OR Computer Reasoning OR Reasoning, Computer OR AI (Artificial Intelligence) OR Computer Vision Systems OR Computer Vision System OR System, Computer Vision OR Systems, Computer Vision OR Vision System, Computer OR Vision Systems, Computer OR Knowledge Acquisition (Computer) OR Acquisition, Knowledge (Computer) OR Knowledge Representation (Computer) OR Knowledge Representations (Computer)) OR ((Representation, Knowledge (Computer)) AND (Neuroblastoma)

**Scopus**

TITLE-ABS-KEY ( radiomics ) OR TITLE-ABS-KEY ( learning, AND machine ) OR TITLE-ABS-KEY ( machine AND learning ) OR TITLE-ABS-KEY ( transfer AND learning ) OR TITLE-ABS-KEY ( learning, AND transfer ) OR TITLE-ABS-KEY ( computational AND intelligence ) OR TITLE-ABS-KEY ( intelligence, AND computational ) OR TITLE-ABS-KEY ( machine AND intelligence ) OR TITLE-ABS-KEY ( intelligence AND machine ) OR TITLE-ABS-KEY ( computer AND reasoning ) OR TITLE-ABS-KEY ( reasoning AND computer ) OR TITLE-ABS-KEY ( ai ) OR TITLE-ABS-KEY ( artificial AND intelligence ) OR TITLE-ABS-KEY ( computer AND vision AND systems ) OR TITLE-ABS-KEY ( computer AND vision AND system ) OR TITLE-ABS-KEY ( system AND computer AND vision ) OR TITLE-ABS-KEY ( systems AND computer AND vision ) OR TITLE-ABS-KEY ( vision AND system AND computer ) OR TITLE-ABS-KEY ( vision AND systems AND computer ) OR TITLE-ABS-KEY ( knowledge AND acquisition AND computer ) OR TITLE-ABS-KEY ( acquisition AND knowledge AND computer ) OR TITLE-ABS-KEY ( knowledge AND representation AND computer ) OR TITLE-ABS-KEY ( knowledge AND representations AND computer ) OR TITLE-ABS-KEY ( representation AND knowledge AND computer ) AND TITLE-ABS-KEY ( neuroblastoma )

**Web of science**

(TS=( radiomics ) OR TS=( learning, AND machine ) OR TS=( machine AND learning ) OR TS=( transfer AND learning ) OR TS=( learning, AND transfer ) OR TS=( computational AND intelligence ) OR TS=( intelligence, AND computational ) OR TS=( machine AND intelligence ) OR TS=( intelligence AND machine ) OR TS=( computer AND reasoning ) OR TS=( reasoning AND computer ) OR TS=( ai ) OR TS=( artificial AND intelligence ) OR TS=( computer AND vision AND systems ) OR TS=( computer AND vision AND system ) OR TS=( system AND computer AND vision ) OR TS=( systems AND computer AND vision ) OR TS=( vision AND system AND computer ) OR TS=( vision AND systems AND computer ) OR TS=( knowledge AND acquisition AND computer ) OR TS=( acquisition AND knowledge AND computer ) OR TS=( knowledge AND representation AND computer ) OR TS=( knowledge AND representations AND computer ) OR TS=( representation AND knowledge AND computer )) AND (TS=( neuroblastoma ))

**PsycInfo**

**Abstract**: Radiomics *OR* **Abstract**: Computational Intelligence *OR* **Abstract**: Computational Intelligence *OR* **Abstract**: Machine Intelligence *OR* **Abstract**: Intelligence, Machine *OR* **Abstract**: Computer Reasoning *OR* **Abstract**: Reasoning, Computer *OR* **Abstract**: AI *OR* **Abstract**: Artificial Intelligence *OR* **Abstract**: Computer Vision Systems *OR* **Abstract**: Computer Vision System *OR* **Abstract**: System, Computer Vision *OR* **Abstract**: Systems, Computer Vision *OR* **Abstract**: Vision System, Computer *OR* **Abstract**: Vision Systems, Computer *OR* **Abstract**: Knowledge Acquisition (Computer) *OR* **Abstract**: (Acquisition, Knowledge (Computer) *OR* **Abstract**: Knowledge Representation (Computer) *OR* **Abstract**: Knowledge Representations (Computer) *OR* **Abstract**: Representation, Knowledge (Computer) *OR* (**Abstract**: Learning, Machine *OR* **Abstract**: Machine Learning *OR* **Abstract**: Transfer Learning *OR* **Abstract**: Learning, Transfer *AND* **Abstract**: Neuroblastoma
