# Supplementary material for: Artificial Intelligence for Diagnosis, Risk Stratification, and Prognosis of Neuroblastoma - A Systematic Review and Meta-Analysis
Source: Curr Oncol Rep. 2026 Sep 4;28(1):90. doi: 10.1007/s11912-026-01824-0 (PMC13545110; doi:10.1007/s11912-026-01824-0)
Supplement: Supplementary file 2 [file 11912_2026_1824_MOESM2_ESM.docx]

Modified QUADAS-II

Risk of bias was assessed using a modified QUADAS-II adapted to AI based prediction models. Modifications were made according to elements from PROBAST (prediction model risk of bias) and CONSORT-AI including feature selection, model validation, and reporting of calibration.

QUADAS-II standard domains (patient selection, index test, reference standard, flow and timing) were maintained, but additional questions within each domain were added according to PROBAST and CONSORT-AI elements.

Phase 1 and 2 were not modified; only Phase 3 was modified. The modifications highlighted in red signal not applicable questions, and those highlighted in blue signal added questions. The rationale for each numbered modification is consolidated in the section below.

Risk of bias judgment followed formal QUADAS-II recommendations of each author independently judging and annotating their justifications. Each study was independently assessed by two reviewers. Disagreements were resolved by consensus. Domain-level judgments were summarized descriptively, and overall study-level risk of bias was determined based on the highest risk domain

Rationale for modifications

The rationales below correspond to the numbered modifications in Phase 3 of the modified QUADAS-II tool. They have been consolidated here to avoid repeating explanatory text throughout the instrument.

**1.1 (Not Applicable) Describe the time interval and any interventions between index test(s) and reference standard:** Rationale: This question was considered to be non applicable to studies of retrospective nature. As their control is composed of predetermined data (be it correct diagnosis, prognosis, etc). And since the reference standard is based on existing data rather than being applied after the AI model, there is no time interval between the index test (AI model) and the reference standard.

**1.2 (Added) Did the study describe its eligibility criteria for participants or the dataset utilized?** Rationale: Adapted from CONSORT-AI item (4a) - Eligibility criteria for participants and 4a (i) Elaboration - State the inclusion and exclusion criteria at the level of participants. It was also adapted from PROBAST question (1.2) Domain 1 (Participants) - 1.2 Were all inclusions and exclusions of participants appropriate?

**1.3 (Added) Did the study describe the settings and locations where the data were collected?** Rationale: Adapted from CONSORT-AI item (4b) Settings and locations where the data were collected.

**1.4 (Added) Was a method used to generate the random allocation sequence?** Rationale: Adapted from CONSORT-AI item (8a) Method used to generate the random allocation sequence and (9) Mechanism used to implement the random allocation sequence (such as sequentially numbered containers), describing any steps taken to conceal the sequence until interventions were assigned.

**1.5 (Added) Did the study describe how the number of samples was calculated?** Rationale: Adapted from CONSORT-AI item (7a) How sample size was determined.

**1.6 (Added) Were predictors defined and assessed in a similar way for all participants?** Rationale: This is a question (2.1) from PROBAST Domain 2 (Predictors).

**2.1 (Reworded) Were the index test results interpreted without knowledge of the results of the reference standard?** Rationale: Reworded to the question “Were predictor assessments made without knowledge of outcome data?”

**2.2 (Added) Does the study describe utilized statistical methods used in analysis?** Rationale: Adapted from CONSORT-AI items (12a) Statistical methods used to compare groups for primary and secondary outcomes and (12b) Methods for additional analyses, such as subgroup analyses and adjusted analyses.

**2.3 (Added) Did the study present a report of features reduction techniques?** Rationale: Feature reduction prevents overfitting, limits data leakage and proxy learning, while also improving transparency and transportability [(1–4)](https://www.zotero.org/google-docs/?UM0aqn).

**2.4 (Added) Were predictor assessments made without knowledge of outcome data?** Rationale: This is a question (2.2) from PROBAST Domain 2 (Predictors).

**2.5 (Added) Were predictors excluded from the outcome definition?** Rationale: This is a question (3.3) from PROBAST Domain 3 (Outcome).

**3.1 (Not Applicable) Were the reference standard results interpreted without knowledge of the results of the index test?** Rationale: The reference standard was defined as the ground truth labels used to evaluate model performance (e.g., histopathological diagnosis, clinical follow-up outcomes, or established clinical criteria) and established independent of models predictions. Therefore in retrospective studies this question was considerable “Not Applicable”.

**3.2 (Added) Was the diagnosis of neuroblastoma confirmed in any way?** Rationale: Confirmation of pathologies diagnosis prevents label bias, proxy learning, ensuring that model performance depends on pathological features [(5–7)](https://www.zotero.org/google-docs/?lBvrCf).

**3.3 (Added) Was the outcome defined and determined in a similar way for all participants?** Rationale: Adapted from CONSORT-AI item (6a) Completely defined pre-specified primary and secondary outcome measures, including how and when they were assessed.

**3.4 (Added) Did the study have Calibration Statistics?** Rationale: Calibration Statistics compares predicted probabilities to observed event and is a core part of prediction-model performance, alongside discrimination and clinical usefulness . It can capture biases that other metrics, such as AUC and accuracy, often hide [(5,8)](https://www.zotero.org/google-docs/?Tx44uA).

**4.1 (Not Applicable) Describe the time interval and any interventions between index test(s) and reference standard:** Rationale: This question was considered to be non applicable to studies of retrospective nature. As their control is composed of predetermined data (be it correct diagnosis, prognosis, etc). And since the reference standard is based on existing data rather than being applied after the AI model, there is no time interval between the index test (AI model) and the reference standard.

**4.2 (Added) Does the study present performance for all models constructed?** Rationale: Adapted from CONSORT-AI items (16) For each group, number of participants (denominator) included in each analysis and whether the analysis was by original assigned groups Outcomes and estimation and (17a) For each primary and secondary outcome, results for each group, and the estimate effect size and its precision (such as 95% confidence interval).

**4.3 (Added) Was a pre-specified or standard outcome definition used?** Rationale: This is a question (3.2) from PROBAST Domain 3 (Outcome).

**4.4 (Added) Was the outcome determined without knowledge of predictor information?** Rationale: This is a question (3.5) from PROBAST Domain 3 (Outcome).

**Phase 1: State the review question:**

Patients (setting, intended use of index test, presentation, prior testing): Index test(s): Reference standard and target condition:

**Phase 2: Draw a flow diagram for the primary study**

**Phase 3: Risk of bias and applicability judgments**

**DOMAIN 1: PATIENT SELECTION**

**A. Risk of Bias**

**Describe methods of patient selection:**

**1.1 Describe the time interval and any interventions between index test(s) and reference standard: (Not Applicable)**

Was a consecutive or random sample of patients enrolled?

Yes/No/**Unclear**

Was a case-control design avoided?

Yes/No/Unclear

Did the study avoid inappropriate exclusions?

Yes/No/**Unclear**

**1.2 Did the study describe its eligibility criteria for participants or the dataset utilized?**

**Yes/No/Unclear**

**1.3 Did the study describe the settings and locations where the data were collected?**

**Yes/No/Unclear**

**1.4 Was a method used to generate the random allocation sequence?**

**Yes/No/Unclear**

**1.5 Did the study describe how the number of samples was calculated?**

Yes/No/Unclear

**1.6 Were predictors defined and assessed in a similar way for all participants?	Yes/No/Unclear**

**Could the selection of patients have introduced bias?**

RISK: LOW/HIGH/UNCLEAR

**B. Concerns regarding applicability**

Describe included patients (prior testing, intended use of index test and setting):

Is there concern that the included patients do not match the review question?

CONCERN: LOW/HIGH/UNCLEAR

**DOMAIN 2: INDEX TEST(S)**

If more than one index test was used, please complete it for each test.

**A. Risk of Bias**

**Describe the index test and how it was conducted and interpreted:**

**2.1 Were the index test results interpreted without knowledge of the results of the reference standard?**

**Yes/No/Unclear**

If a threshold was used, was it pre-specified?

Yes/No/Unclear

**2.2 Does the study describe utilized statistical methods used in analysis?**

**Yes/No/Unclear**

**2.3 Did the study present a report of features reduction techniques?**

**Yes/No/Unclear**

**2.4 Were predictor assessments made without knowledge of outcome data?**

**Yes/No/Unclear**

**2.5 Were predictors excluded from the outcome definition?**

**Yes/No/Unclear**

**Could the conduct or interpretation of the index test have introduced bias?**

RISK: LOW /HIGH/UNCLEAR

**B. Concerns regarding applicability**

Is there concern that the index test, its conduct, or interpretation differ from the review question?

CONCERN: LOW /HIGH/UNCLEAR

**DOMAIN 3: REFERENCE STANDARD**

**A. Risk of Bias**

**Describe the reference standard and how it was conducted and interpreted:**

**3.1 Were the reference standard results interpreted without knowledge of the results of the index test? (Not Applicable)**

**Yes/No/Unclear**

Is the reference standard likely to correctly classify the target condition?

Yes/No/Unclear

**3.2 Was the diagnosis of neuroblastoma confirmed in any way?**

**Yes/No/Unclear**

**3.3 Was the outcome defined and determined in a similar way for all participants?**

**Yes/No/Unclear**

**3.4 Did the study have Calibration Statistics?**

**Yes/No/Unclear**

**Could the reference standard, its conduct, or its interpretation have introduced bias?**

RISK: LOW /HIGH/UNCLEAR

**B. Concerns regarding applicability**

Is there concern that the target condition as defined by the reference standard does not match the review question?

CONCERN: LOW /HIGH/UNCLEAR

**DOMAIN 4: FLOW AND TIMING**

**A. Risk of Bias**

Describe any patients who did not receive the index test(s) and/or reference standard or who were excluded:

**4.1 Describe the time interval and any interventions between index test(s) and reference standard: (Not applicable)**

Did all patients receive a reference standard?

Yes/No/**Unclear**

Did patients receive the same reference standard?

Yes/No/**Unclear**

Were all patients included in the statistical analysis?

Yes/No/**Unclear**

**4.2 Does the study present performance for all models constructed?**

**Yes/No/Unclear**

**4.3 Was a pre-specified or standard outcome definition used?**

**Yes/No/Unclear**

**4.4 Was the outcome determined without knowledge of predictor information?**

**Yes/No/Unclear**

**Could the patient flow have introduced bias?**

RISK: LOW /HIGH/UNCLEAR

References

[1.](https://www.zotero.org/google-docs/?0gMeKU)	[Ziegler A, König I. Vollständiges und präzises Berichten von Studien zur diagnostischen Genauigkeit: Die STARD-Initiative. DMW - Dtsch Med Wochenschr. 2011 Feb;136(08):e16–9. doi:10.1055/s-0031-1272979](https://www.zotero.org/google-docs/?0gMeKU)

[2.](https://www.zotero.org/google-docs/?0gMeKU)	[Korevaar DA, Cohen JF, Reitsma JB, Bruns DE, Gatsonis CA, Glasziou PP, et al. Updating standards for reporting diagnostic accuracy: the development of STARD 2015. Res Integr Peer Rev. 2016 Dec;1(1):7. doi:10.1186/s41073-016-0014-7](https://www.zotero.org/google-docs/?0gMeKU)

[3.](https://www.zotero.org/google-docs/?0gMeKU)	[Wolff RF, Moons KGM, Riley RD, Whiting PF, Westwood M, Collins GS, et al. PROBAST: A Tool to Assess the Risk of Bias and Applicability of Prediction Model Studies. Ann Intern Med. 2019 Jan 1;170(1):51–8. doi:10.7326/M18-1376 PubMed PMID: 30596875.](https://www.zotero.org/google-docs/?0gMeKU)

[4.](https://www.zotero.org/google-docs/?0gMeKU)	[Moons KGM, Wolff RF, Riley RD, Whiting PF, Westwood M, Collins GS, et al. PROBAST: A Tool to Assess Risk of Bias and Applicability of Prediction Model Studies: Explanation and Elaboration. Ann Intern Med. 2019 Jan 1;170(1):W1–33. doi:10.7326/M18-1377](https://www.zotero.org/google-docs/?0gMeKU)

[5.](https://www.zotero.org/google-docs/?0gMeKU)	[Sounderajah V, Ashrafian H, Rose S, Shah NH, Ghassemi M, Golub R, et al. A quality assessment tool for artificial intelligence-centered diagnostic test accuracy studies: QUADAS-AI. Nat Med. 2021 Oct;27(10):1663–5. doi:10.1038/s41591-021-01517-0](https://www.zotero.org/google-docs/?0gMeKU)

[6.](https://www.zotero.org/google-docs/?0gMeKU)	[Seyyed-Kalantari L, Zhang H, McDermott MBA, Chen IY, Ghassemi M. Underdiagnosis bias of artificial intelligence algorithms applied to chest radiographs in under-served patient populations. Nat Med. 2021 Dec;27(12):2176–82. doi:10.1038/s41591-021-01595-0](https://www.zotero.org/google-docs/?0gMeKU)

[7.](https://www.zotero.org/google-docs/?0gMeKU)	[Researchers Discover Bias in AI Models That Analyze Pathology Samples | Harvard Medical School [Internet]. 2025 [cited 2026 May 28]. Available from: https://hms.harvard.edu/news/researchers-discover-bias-ai-models-analyze-pathology-samples](https://www.zotero.org/google-docs/?0gMeKU)

[8.](https://www.zotero.org/google-docs/?0gMeKU)	[Performance Metrics for the Comparative Analysis of Clinical Risk Prediction Models Employing Machine Learning. Circ Cardiovasc Qual Outcomes [Internet]. [cited 2026 May 28]. Available from: https://www.ahajournals.org/doi/10.1161/CIRCOUTCOMES.120.007526](https://www.zotero.org/google-docs/?0gMeKU)
