# Supplementary material for: Artificial Intelligence for Diagnosis, Risk Stratification, and Prognosis of Neuroblastoma - A Systematic Review and Meta-Analysis
Source: Curr Oncol Rep. 2026 Sep 4;28(1):90. doi: 10.1007/s11912-026-01824-0 (PMC13545110; doi:10.1007/s11912-026-01824-0)
Supplement: Supplementary file 3 [file 11912_2026_1824_MOESM5_ESM.docx]

Forest Plots

The forest of the meta analysis´ six outcomes are showcased here. The title and parameter used are in the title and below is their respective forest plot. Additionally, the two types of analysis: Formal Meta-Analysis (FMA) and Descriptive inverse-variance summary (DIVS) marks which were adopted according to the outcome. They also differ in plot visualization to facilitate distinction.

(FMA) Differentiation from other Diseases (AUC)

(FMA) Risk Stratification (AUC)

(DIVS) Pathology (AUC)

**(DIVS) Chemotherapy Response (AUC)**

(FMA) Metastasis Response (AUC)

**(DIVS) Sensitivity (Internal vs External Validation) of HN models**

**(DIVS) Specificity (Internal vs External Validation of HN models)**

**(DIVS) Accuracy (Internal vs External validation) of HN models**

(FMA) MYCN (AUC)

**(DIVS) Accuracy (AUC)**
